# Study Profile of the Tsuruoka Metabolomics Cohort Study (TMCS)

**DOI:** 10.2188/jea.JE20230192

**Published:** 2024-08-05

**Authors:** Sei Harada, Miho Iida, Naoko Miyagawa, Aya Hirata, Kazuyo Kuwabara, Minako Matsumoto, Tomonori Okamura, Shun Edagawa, Yoko Kawada, Atsuko Miyake, Ryota Toki, Miki Akiyama, Atsuki Kawai, Daisuke Sugiyama, Yasunori Sato, Ryo Takemura, Kota Fukai, Yoshiki Ishibashi, Suzuka Kato, Ayako Kurihara, Mizuki Sata, Takuma Shibuki, Ayano Takeuchi, Shun Kohsaka, Mitsuaki Sawano, Satoshi Shoji, Yoshikane Izawa, Masahiro Katsumata, Koichi Oki, Shinichi Takahashi, Tsubasa Takizawa, Hiroshi Maruya, Yuji Nishiwaki, Ryo Kawasaki, Akiyoshi Hirayama, Takamasa Ishikawa, Rintaro Saito, Asako Sato, Tomoyoshi Soga, Masahiro Sugimoto, Masaru Tomita, Shohei Komaki, Hideki Ohmomo, Kanako Ono, Yayoi Otsuka-Yamasaki, Atsushi Shimizu, Yoichi Sutoh, Atsushi Hozawa, Kengo Kinoshita, Seizo Koshiba, Kazuki Kumada, Soichi Ogishima, Mika Sakurai-Yageta, Gen Tamiya, Toru Takebayashi

**Affiliations:** 1 Department of Preventive Medicine and Public Health, Keio University School of Medicine, Tokyo, Japan; 2 Institute for Advanced Biosciences, Keio University, Yamagata, Japan; 3 Department of Obstetrics and Gynecology, Keio University School of Medicine, Tokyo, Japan; 4 Faculty of Environment and Information Studies, Keio University, Kanagawa, Japan; 5 Faculty of Nursing and Medical Care and Graduate School of Health Management, Keio University, Kanagawa, Japan; 6 Biostatistics Unit, Clinical and Translational Research Center, Keio University Hospital, Tokyo, Japan; 7 Department of Cardiology, Keio University School of Medicine, Tokyo, Japan; 8 Duke Clinical Research Institute, Durham, NC, USA; 9 Department of Neurology, Keio University School of Medicine, Tokyo, Japan; 10 Department of Neurology, Tokyo Saiseikai Central Hospital, Tokyo, Japan; 11 Department of Neurology and Stroke, Saitama Medical University International Medical Center, Saitama, Japan; 12 Shonai Hospital, Yamagata, Japan; 13 Department of Environmental and Occupational Health, School of Medicine, Toho University, Tokyo, Japan; 14 Division of Public Health, Department of Social Medicine, Graduate School of Medicine, Osaka University, Osaka, Japan; 15 Division of Biomedical Information Analysis, Institute for Biomedical Sciences of Iwate Medical University, Iwate, Japan; 16 Division of Biomedical Information Analysis, Iwate Tohoku Medical Megabank Organization, Disaster Reconstruction Center, Iwate Medical University, Iwate, Japan; 17 Tohoku Medical Megabank Organization, Tohoku University, Sendai, Japan; 18 Graduate School of Medicine, Tohoku University, Sendai, Japan; 19 Graduate School of Information Sciences, Tohoku University, Sendai, Japan; 20 Advanced Research Center for Innovations in Next-Generation Medicine, Tohoku University, Sendai, Japan; 21 Institute of Development, Aging and Cancer, Tohoku University, Sendai, Japan; 22 Center for Advanced Intelligence Project, RIKEN, Tokyo, Japan

**Keywords:** cohort study, metabolomics, multi-omics, aging

## Abstract

The Tsuruoka Metabolomics Cohort Study (TMCS) is an ongoing population-based cohort study being conducted in the rural area of Yamagata Prefecture, Japan. This study aimed to enhance the precision prevention of multi-factorial, complex diseases, including non-communicable and aging-associated diseases, by improving risk stratification and prediction measures. At baseline, 11,002 participants aged 35–74 years were recruited in Tsuruoka City, Yamagata Prefecture, Japan, between 2012 and 2015, with an ongoing follow-up survey. Participants underwent various measurements, examinations, tests, and questionnaires on their health, lifestyle, and social factors. This study uses an integrative approach with deep molecular profiling to identify potential biomarkers linked to phenotypes that underpin disease pathophysiology and provide better mechanistic insights into social health determinants. The TMCS incorporates multi-omics data, including genetic and metabolomic analyses of 10,933 participants, and comprehensive data collection ranging from physical, psychological, behavioral, and social to biological data. The metabolome is used as a phenotypic probe because it is sensitive to changes in physiological and external conditions. The TMCS focuses on collecting outcomes for cardiovascular disease, cancer incidence and mortality, disability and functional decline due to aging and disease sequelae, and the variation in health status within the body represented by omics analysis that lies between exposure and disease. It contains several sub-studies on aging, heated tobacco products, and women’s health. This study is notable for its robust design, high participation rate (89%), and long-term repeated surveys. Moreover, it contributes to precision prevention in Japan and East Asia as a well-established multi-omics platform.

## PURPOSE

The rationale of the Tsuruoka Metabolomics Cohort Study (TMCS) is to achieve precision prevention of multi-factorial, complex diseases, including non-communicable and aging-associated diseases and conditions, through building better risk stratification and prediction measures. A cohort study that employs an integrative approach with deep molecular profiling represents a promising epidemiological approach to achieve this aim. Previous epidemiological studies have mainly focused on genomic information, and large-scale genome-wide association analyses have identified novel genetic loci. However, genes alone account for the minority of disease etiology for most chronic diseases and conditions, while lifestyle and environmental risk factors play critical roles in their development. This demonstrates the need for quantifying the cumulative impact of these factors alongside their interactions with the genetic background.^1^

Therefore, we initiated a population-based cohort study incorporating multi-omics data. The use of integrative omics approaches, such as gene–environment interaction or gene–microbiome–environment interaction, allows us to identify potential biomarkers linked to phenotypes that underpin the pathophysiology of diseases and will provide a better phenotypic understanding of diseases and better mechanistic insights into social determinants of health. Accordingly, the enhancement of precise risk-stratification and prediction modeling will yield benefits, not only at the individual level, but also in its applicability to the population level. Applying multi-omics techniques to epidemiology will also refine health risk assessment for emerging environmental and public health issues, proposed as ‘meet-in-the-middle’ approach^2^^–^^4^ or ‘exposome’ concept.^5^

## MAIN FEATURES

TMCS is the sole study designed specifically for conducting an integrative, longitudinal evaluation of gene–environment interactions in Japan. It distinguishes itself by utilizing extensive metabolomic measurements in a population-based cohort study including over 10,000 participants in Japan. The study is also notable in terms of its robust design; high participation rate (89%) from the regional population; comprehensive data collection, ranging from physical, psychological, behavioral, and social to biological data with multi-omics (genetic analysis of over 10,000 participants and DNA methylation of over 3,000 participants); and long-term repeated surveys. Metabolome is a useful probe of phenotypes, as they are sensitive to changes in both physiological and external conditions as the downstream products of multiple biological processes of genes, transcriptional factors, and proteins.^6^ By integrating metabolomics data along with other omics information and associations between risk factors and disease, the TMCS can unravel the “meet-in-the-middle” concept or the link between exposure and outcome, which can be elucidated along with in vivo mechanisms.^5^ Prior to the baseline survey (wave 1), we identified both biological and analytical factors that could affect metabolomic data,^1^ thereby establishing a standardized protocol for obtaining unbiased profiles through multiple runs over a long period of time.^7^ It is also promising for improving dietary assessment in nutritional epidemiology,^8^ as profiles of urinary metabolites may be used as proxy measurements to understand functional relationships between nutrients and health outcomes.^9^

The TMCS particularly focuses on collecting outcomes for cardiovascular disease (CVD), stroke, cancer incidence and mortality, disability and functional decline due to aging and disease sequelae, and the variation in health status within the body represented by omics analysis between exposure and disease. It contains several sub-studies, including those on aging, heated tobacco products (HTP), and women’s health. The aging sub-study longitudinally assessed the six key domains of intrinsic capacity proposed by the World Health Organization^10^ along with multi-omics data.

HTP has been highlighted as a public health issue that urgently needs to be addressed since its market launch in Japan in 2014. Evidence of the health effects of the continued use of HTPs is lacking. Nonetheless, traditional epidemiological methods require a remarkably long time to assess their impact on the primary outcomes of cancer and heart disease. The HTP sub-study aims to investigate early health effects of HTP through multi-omics analysis and establish a method to rapidly assess the health effects of such new exposures using omics data by employing this as a model case.

Advanced research on women’s health and sex-related differences is another key element. Sex-related differences characterize many health conditions; however, research remains underexplored. Furthermore, reproductive health is increasingly recognized as an important factor in shaping the overall well-being of women. However, the impact of genetic factors and lifestyle on reproductive health is yet to be elucidated. The use of multi-omics data is expected to advance our understanding of sex-related differences in health and their underlying mechanisms.

Using these data, we aim to unravel connections between longitudinal aging processes at the biological, phenotypic, and functional levels and contribute to the elucidation of the ongoing process of developing and maintaining the functional ability that portends well-being in older age.^11^

## PARTICIPANTS

**Figure 1.**
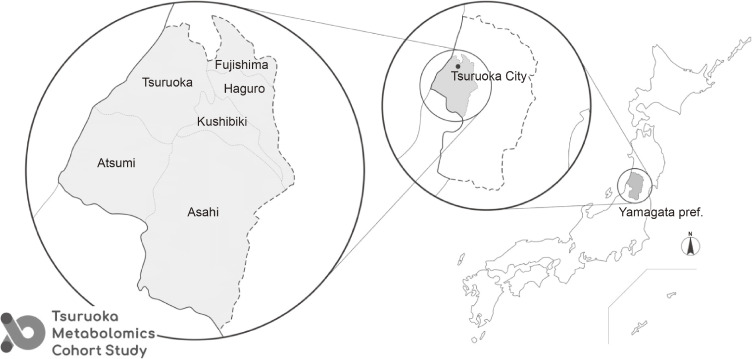
Study area of the Tsuruoka Metabolomics Cohort Study

**Figure 2.**
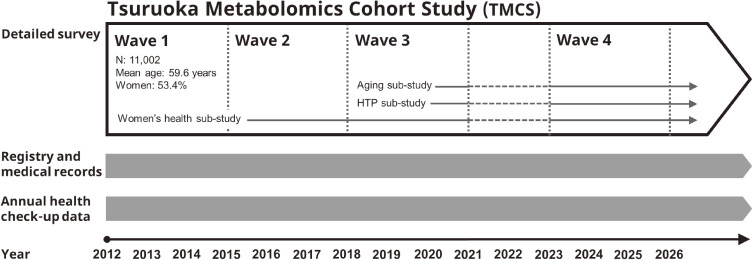
Time course of the Tsuruoka Metabolomics Cohort Study. HTP, heated tobacco products.

Residents and workers of Tsuruoka City, Yamagata Prefecture, located in the Tohoku region of Japan, aged 35–74 years, were eligible for this study (Figure 1). During the 3-year baseline survey (Wave 1) between April 2012 and March 2015, 11,002 participants (5,131 men and 5,871 women; age, 59.6 [standard deviation {SD}, 10.1] years) were recruited from a total of 12,332 recipients of health check-up programs provided by the city of Tsuruoka or employers (Figure 2). There were 8,428 participants (4,017 men and 4,411 women; age, 63.3 [SD, 7.5] years) from a municipal health check-up and 2,574 participants (1,114 men and 1,460 women; age, 47.7 [SD, 7.9] years) from a worksite health check-up (eFigure 1). Combining these two recruitment strategies ensures greater regional representation. All participants underwent blood and urine metabolome measurements, physical examinations, laboratory tests, and completed detailed questionnaires on health status, lifestyle, and social factors. The study base of the three aforementioned sub-studies was the same as that of the cohort. Moreover, 47 participants (12 men and 35 women; age, 73.7 [SD, 4.5] years) and 46 participants (28 men and 18 women; age, 35.8 [SD, 3.6] years) were additionally recruited for the Wave 3 survey for the aging and HTP sub-studies, respectively. At the time of Wave 3, the number of the aging sub-study participants was 2,601 and the total number of the HTP sub-study participants was 6,050.

The study protocol was approved by the Medical Ethics Committee of the Keio University School of Medicine, Tokyo, Japan (Approval Nos. 20110264, 20170300, 20180207, 20180336, and 20190172). All the participants provided written informed consent.

## OUTCOMES AND FOLLOW-UP

Table 1 lists the major hard and soft outcomes obtained in TMCS. One of the primary targets is CVD incidence. Data on first-episode acute coronary syndromes and stroke were collected through medical record surveys.^12^ Specifically, comprehensive surveys of medical records from five core hospitals in the region were conducted annually. The selected hospitals provided complete coverage of the area where TMCS participants would receive acute care management, ensuring the inclusiveness of the survey. The final outcome decisions were made by a panel of experienced physicians, including at least two cardiologists and neurologists, based on the symptoms, electrocardiography (ECG), coronary angiography, computed tomography (CT), and/or magnetic resonance imaging (MRI), and/or magnetic resonance angiography (MRA). Cancer data were collected from official cancer registries in Japan and Yamagata Prefecture. Mortality data, including causes of death, were collected from the public death certificates of all the deceased participants with the approval of the Ministry of Health, Labour and Welfare. Disability outcomes were obtained from the national long-term care insurance system, which provides information on the nursing care level based on the assessment of care requirements for participants over the age of 65 years. Level of support was used to assess the disability outcome. In addition, data on health insurance coverage were obtained, which allowed access to data on the medication costs to be used as outcomes of the study. Assessing the prodromal phases of the disease as outcomes is equally important to capture detailed changes in health status. These include hypertension, lipid abnormalities, diabetes, use of new medications, arrhythmias, visceral fat accumulation, subclinical atherosclerosis indices, decreased function and failure of various organs, and mental health issues. Identifying the metabolic changes that occur between exposure and disease allows us to epidemiologically elucidate the disease mechanisms.

**Table 1.** Hard and soft outcomes identified from resource use and health assessments

List of outcomes	TMCS	Aging sub-study	HTP sub-study	Women’s health sub-study
Registry and medical record survey^a^				
Mortality	Annual	Annual	Annual	Annual
Cardiovascular diseases (acute coronary syndromes and stroke)	Annual	Annual	Annual	Annual
Cancer	Annual	Annual	Annual	Annual
Disability	Annual	Annual	Annual	Annual
Medication	Annual	Annual	Annual	Annual
Annual health check-up data^b^				
Hypertension, lipid abnormalities, diabetes	Annual	Annual	Annual	Annual
Detailed survey^c^				
Arrhythmia, visceral fat accumulation, subclinical atherosclerosis factors	Wave 1–4	Wave 1–4	Wave 1–4	Wave 1–4
Decreased function and failure of various organs (liver, kidney, lung, sensory organ)	Wave 1–4	Wave 3, 4	Wave 3, 4	Wave 1–4
Mental health (depression, stress, insomnia)	Wave 1–4	Wave 3, 4	Wave 3, 4	Wave 1–4
Reproductive health (menopausal disorder)	Wave 1–4	—	—	Wave 1–4
Osteoporosis	Wave 1–4	Wave 1–4	—	Wave 1–4
Frailty (physical-, social-, mental-)	Wave 1–4	Wave 3, 4	—	—
IADLs, ADLs	Wave 3, 4	Wave 3, 4	—	—
Cognitive function (dementia, MCI)	—	Wave 3, 4	—	—
Brain MRI (atrophy, atherosclerosis)	—	Wave 3, 4	—	—
Molecular profiles				
Metabolome (blood and urine)	Wave 1–4	Wave 3, 4	Wave 3, 4	Wave 1–4
DNA methylation and gene expression	Wave 3, 4	—	Wave 3, 4	—
Biological age by DNA methylation analysis	Wave 3, 4	Wave 3, 4	—	—

ADLs, activities of daily living; HTP, heated tobacco products; IADLs, instrumental activities of daily living; MCI, mild cognitive impairment; MRI, magnetic resonance imaging; TMCS, Tsuruoka Metabolomics Cohort Study.

^a^Registry and medical record survey was conducted annually. Mortality data were collected from the public death certificates of all the deceased participants with the approval of the Ministry of Health, Labour and Welfare. Data on first-episode acute coronary syndromes and stroke were collected through medical record surveys. Cancer data were collected from official cancer registries in Japan and the Yamagata Prefecture. Disability outcomes were obtained from the national long-term care insurance system. Medication data were obtained from health claims data.

^b^Annual health check-up data were provided by the municipality, employers, or employment-based insurers, which allowed us to track changes on a yearly basis.

^c^Detailed surveys were conducted every 3–5 years, which were referred to as waves 1, 2, 3, and 4 (Figure 2).

These outcome data were collected through follow-up surveys conducted from 2015 onwards, which were referred to as waves 2, 3, and 4 (Figure 2), as well as from the annual health check-ups provided by the municipality, employers, or employment-based insurers, which allowed us to track changes on a yearly basis. In the aging sub-study, physical frailty, social frailty, and osteoporosis were assessed in waves 3 and 4. Brain MRI and cognitive function tests were also conducted on a subset of participants; mental health, nutrition, swallowing function, and instrumental activities of daily living (IADLs) were assessed through questionnaires, and biological age was assessed through DNA methylation analysis. For the HTP sub-study, comprehensive testing of DNA methylation and gene expression was conducted from wave 3 onwards. The women’s health sub-study has been ongoing since wave 1, and outcomes related to reproductive health, as well as osteoporosis and cognitive impairment, have been evaluated, some of which are common outcomes with the aging sub-study, yet the focus is specifically on age-related sex differences.

## MEASUREMENTS

Table 2 summarizes the phenotypic data collected from the TMCS and timing. The following sections provide details of the phenotypic data.

**Table 2.** Phenotypic data collected in the Tsuruoka Metabolomics Cohort Study

Phenotypic data	Examples	Wave 1	Wave 2	Wave 3^a^	Wave 4^a^	Annual health check-ups
Physical examinations						
Obesity	Anthropometric	X	X	X	X	X
	Abdominal fat (impedance)	X	X	X	—	—
Cardiovascular	Blood pressure	X	X	X	X	X
	Electrocardiogram	X	X	X	X	—
	CAVI^b^, ABI^b^	X	—	—	—	—
Sensory receptor	Eye examination, hearing test	X	X	X	X	—
Pulmonary function tests		X	X	X	X	—
Laboratory						
Blood	CBC, lipids, glucose, hemoglobin A1c, creatinine, hepatic enzymes	X	X	X	X	X
	Uric acid, BUN, protein, albumin, BNP, IRI	X	X	X	X	—
	Cystatin C^b^	X	—	X	—	—
Urine	Urinalysis	X	X	X	X	—
	Sodium, potassium	X	X	X	X	—
	Microalbumin^b^	X	—	X	—	—
	NAG^b^	X	—	—	—	—
Imaging tests						
	Chest X-rays	X	X	X	X	—
	Non-mydriatic fundus examination	X	—	X	X	—
	AMD diagnosis/grading	X	—	X	X	—
	Abdominal ultrasound^b^	X	X	X	X	—
	Abdominal fat (CT)	—	—	—	X	—
	Brain MRI (atrophy, atherosclerosis)^b^	—	—	X	X	—
	Bone mineral density (DXA)	X	—	X	X	—
Physical performance tests						
	SPPB (hand grip, 4m-walking speed, chair stand, balance test)	—	—	X	X	—
	Body composition (skeletal muscle mass, fat mass) (BIA)	—	—	X	X	—
Cognitive function tests						
	Neuropsychological tests (MMSE, MoCA-J, WAIS-IV)	—	—	X	X	—
	Physician consultation and diagnosis (dementia/MCI/normal)	—	—	X	X	—
Interview and questionnaire						
Lifestyle	Smoking status, alcohol intake, physical activity	X	X	X	X	X
	Sleep (quality and quantity)	X	—	X	X	—
	Dietary habit (food and nutrition intake)	X	—	X	X	—
	Dietary supplement use	X	X	X	X	—
	Passive smoking	X	—	X	X	—
	Heated tobacco products	—	—	X	X	—
Social factors:	Social network	X	—	X	X	—
	Social support	X	—	—	—	—
	Social participation	X	—	X	X	—
Psychosocial factors	Educational attainment, occupation status	X	—	X	X	—
	Marital status	X	X	X	X	—
	Family and household composition	X	—	X	X	—
Medication and supplement use		X	X	X	X	—
Health status	Medical history	X	X	X	X	X
	Family history	X	—	—	—	—
	Subjective Happiness	X	—	—	—	—
	Stress coping	X	—	—	—	—
	Self-efficacy	—	—	X	—	—
	Job strain	X	—	X	X	—
	Depression	X	—	X	X	—
	Women’s reproductive health	X	X	X	X	—
	Activities of daily living, fractures, osteoporosis, osteoarthritis	—	—	X	X	—
	Oral health (number of teeth, swallowing function)	—	—	X	X	—
Metabolome						
Blood	Charged metabolites	X	X	X	X	—
	Lipids	X	X	X	X	—
Urine	Charged metabolites	X	X	X	X	—
Genomics and epigenomics						
	Genome	X	—	X	—	—
	DNA methylation & gene expression^b^	—	—	X	X	—
	Epigenetic clocks^b^	—	—	X	X	—
Other biomarker measurements						
Blood	Inflammatory, Immune	X	X	X	X	—
	Lipid subfractions and characteristics, LAA, LAB, adiponectin, hormones and vitamins	X	—	X	X	—
Urine	Environmental chemicals^b^	X	—	X	—	—
	Cotinine^b^	—	—	X	X	—

ABI, ankle-brachial index; BUN, blood urea nitrogen; AMD, age-related macular degeneration; BIA, bioelectrical impedance analysis; BNP, brain natriuretic peptide; CAVI, cardio-ankle vascular index; CBC, complete blood count; CT, Computed Tomography; DXA, Dual-energy X-ray absorptiometry; ECG, electrocardiogram; HTP, heated tobacco products; IRI, immunoreactive insulin; LAA, lectin-like oxidized low-density lipoprotein receptor-1 ligand containing apo AI; LAB, lectin-like oxidized low-density lipoprotein receptor-1 ligand containing apo B; MCI, mild cognitive impairment; MMSE, mini-mental state examination; MoCA-J, Japanese version of the Montreal Cognitive Assessment; NAG, N-acetyl-β-D-Glucosaminidase; SPPB, short physical performance battery; WAIS-IV, Wechsler adult intelligence scale IV.

^a^Aging and HTP substudies included.

^b^Measured on selected participants.

### Physical examinations, laboratory tests, and imaging tests

Measurements of obesity (anthropometry, abdominal fat), CVD risk factors (blood pressure, ECG, cardio-ankle vascular index [CAVI],^13^ and ankle-brachial index [ABI]), sensory function (corrected visual acuity and hearing acuity), and pulmonary function tests^14^ were collected through physical examinations. Systolic and diastolic blood pressure was measured twice after resting using an automated sphygmomanometer (OMRON HBP-T105S-N; OMRON Corporation, Kyoto, Japan), and the average of the two measurements was used.

Common laboratory tests were conducted on blood and urine samples, including complete blood count, chemistry panel, lipid and glucose profiles, urinalysis, and urine sodium and potassium concentrations. Additional measurements included cystatin C in the blood and microalbumin and N-acetyl-beta-D-glucosaminidase (NAG) in the urine of a subset of participants. Each test was conducted using the methods standardized by the Japanese Committee for Clinical Laboratory Standards.^15^

Chest radiography and fundus examinations (non-mydriatic) were performed for all participants. The diagnosis and grading of age-related macular degeneration (AMD) were conducted by ophthalmologists based on the fundus images.^16^ Other imaging tests included abdominal ultrasound, CT of abdominal fat, brain MRI, and bone mineral density using dual-energy X-ray absorptiometry.

### Physical performance and cognitive function tests

The Short Physical Performance Battery (SPPB) test,^17^ a composite measure assessing walking speed, standing balance, sit-to-stand performance, and hand grip strength, was conducted in an aging sub-study population. Objective assessments of skeletal muscle and fat masses were also performed using a multi-frequency bioelectrical impedance device.^18^ Trained clinical psychologists assessed the cognitive function through face-to-face interviews, and the Mini-Mental State Examination (MMSE)^19^ and the Japanese version of the Montreal Cognitive Assessment (MoCA-J)^20^^,^^21^ were used to screen for cognitive impairment. Those with scores below the screening threshold were examined by a physician specializing in dementia management.

### Interviews and questionnaires

Structured questionnaires were used to assess lifestyle, social factors, medication and supplement use, and various health statuses, including medical and family histories. Face-to-face interviews were conducted to avoid missing responses. The validity of diet, physical activity, and self-reported medication use were assessed using the weighing method,^22^^,^^23^ accelerometers,^24^ and health insurance claims data,^25^ respectively.

### Biological sample collection and storage

Plasma, serum, buffy coat, and urine samples were collected in the morning between 8:30 and 10:30 am after overnight fasting to avoid variations due to fasting and circadian rhythms. Plasma samples were collected with EDTA-2Na as an anticoagulant and kept at 4°C immediately after collection.

### Metabolomics measurement

Non-targeted mass spectrometry-based metabolomic profiling was performed using fasting plasma samples via capillary electrophoresis time-of-flight mass spectrometry (CE-TOF-MS). Metabolite extraction from plasma was completed within 6 hours after collection to minimize the effect of metabolic changes in the plasma.^26^ The extraction method has been previously described in detail.^27^ The CE-TOF-MS analysis of cationic and anionic metabolites was performed as previously described.^27^ Raw data were processed using our proprietary software (MasterHands).^28^^,^^29^ As a preliminary study, we identified 290 metabolite peaks (131 cations and 159 anions) in the plasma: 154 known with standard compounds and 136 unknown. We decided to measure a priori the absolute concentrations of the 115 metabolites (63 cations and 52 anions) that were expected to be stably observed in most human plasma samples and matched with standard compounds. We used two fixed CE-TOF-MS instruments to exclusively measure the cations and anions. Mass calibration using the tuning solution and MS entrance cleaning were performed at the beginning of every batch to ensure robust performance. To avoid unexpected changes in the sensitivity or variance of the mass measurement during a continuous run, the number of samples per batch was limited to 100. To monitor the metabolome analysis stability, quality control (QC) samples were injected every 10 samples and assessed at the start of the analytical run and at intervals throughout the analysis. Moreover, subsequent samples were re-analyzed when the concentration of each metabolite in the QC samples continuously exceeded twice the value of the mean concentration ± two standard deviations for more than half the metabolites. Details of the evaluation studies for the reproducibility and validity of CE-TOF-MS measurements have been previously reported for plasma^26^ and urine.^30^

Targeted lipid analyses in plasma were performed using liquid chromatography-tandem mass spectrometry (LC-MS/MS). We selected 147 clinically important lipids, including bioactive lipids and lipid mediators. The sample preparation protocol and analytical method were previously described in detail.^31^ Each batch consisted of 40 samples, and the QC samples were measured at the beginning and end of each batch, and for every 10 samples. The mass spectrometer underwent maintenance once a year and was calibrated every 3 months. The following strategies were adopted for data normalization. First, the median value of each lipid was calculated from the quantitative values of the QC samples measured in the same batch. Next, the lipid concentration in each sample was quantified. Finally, the normalized values were calculated by dividing the concentration by the median value of the QC samples. Details of the method validation and data stability have been previously reported.^31^

### Genotyping and imputation

Genotyping was conducted using the Japonica Array NEO,^32^ a custom array containing 666,883 markers designed based on an allele frequency panel of 3,552 Japanese individuals, 3.5KJPNv2.^33^ The genotyping experiments were carried out according to the manufacturer’s protocol, and further details have been previously described.^32^ The genotype calling was performed in two separate batches. Quality control analyses were conducted to exclude low-quality samples with low call rates, variants with high missing rates, significant deviations from the Hardy–Weinberg equilibrium, or low minor allele frequency. To obtain an imputed dataset, pre-phasing was conducted using SHAPEIT2.^34^ The imputation was performed with IMPUTE4^35^ using a cross-imputed haplotype reference panel consisting of 3.5KJPNv2^33^ and 1KGP Phase 3.^36^

### DNA methylation (DNAm) and gene expression analyses

For the HTP sub-study, blood-derived DNA and RNA were used for the following omics analyses: (1) DNAm analysis using pyrosequencing (QIAGEN) on well-known DNAm biomarkers (17 genes and 29 CpGs) for combustible tobacco smoking^37^; (2) Comprehensive DNAm analysis using the MethylationEPIC microarray (Illumina) and epigenome-wide association analysis of smoking habits; and (3) Comparison of gene expression among smoking habits using RNA sequencing.^37^ For the aging sub-study, DNAm analysis using CDMV-seq (Common DNA Methylation Variation sequencing^38^) on blood-derived DNA and epigenetic age will be conducted using the sequencing-based epigenetic age calculation algorithm developed by Komaki et al.^39^

## BASELINE CHARACTERISTICS

In total, 11,002 individuals participated in this study (46.6% men and 53.3% women). The distribution of age at baseline was 60.0 (SD, 9.9) years for men and 59.3 (SD, 10.2) years for women. The smoking rate was 27.5% for men and 4.3% for women, both of which were slightly lower than the national average (men in 40s: 43.2%, 50s: 41.0%, 60s: 31.9%, women in 40s: 12.7%, 50s: 11.9%, 60s: 8.0%).^40^ The habitual drinking rate was higher than the national average, especially among men (75.8% for men and 28.1% for women). The prevalence of hypertension and diabetes tended to be lower than the national average^40^ (Table 3).

**Table 3.** Characteristics of the participants in the Tsuruoka Metabolomics Cohort Study

	Total	Men	Women
Number	11,002	5,131	5,871
Age, years	59.6 (10.1)	60.0 (9.9)	59.3 (10.2)
Age categories			
35–44 years	11.7 (1,288)	11.0 (566)	12.3 (722)
45–54 years	16.2 (1,784)	15.6 (801)	16.7 (983)
55–64 years	33.5 (3,682)	33.2 (1,701)	33.7 (1,981)
65–74 years	38.6 (4,248)	40.2 (2,063)	37.2 (2,185)
Educational attainment			
≤12 years	69.1 (7,602)	71.4 (3,665)	67.1 (3,937)
>12 years	30.5 (3,360)	28.2 (1,445)	32.6 (1,915)
Missing	0.4 (40)	0.4 (21)	0.3 (19)
Working status			
Full-time employed	25.9 (2,853)	26.8 (1,373)	25.2 (1,480)
Part-time employed	11.0 (1,212)	7.7 (395)	13.9 (817)
Self-employed	30.5 (3,361)	40.0 (2,052)	22.3 (1,309)
Not working	32.3 (3,549)	25.2 (1,293)	38.4 (2,256)
Missing	0.2 (27)	0.4 (18)	0.2 (9)
Smoking status			
Never	55.8 (6,143)	20.6 (1,056)	86.6 (5,087)
Former	28.9 (3,178)	51.7 (2,653)	8.9 (525)
Current	15.1 (1,662)	27.5 (1,412)	4.3 (250)
Missing	0.2 (19)	0.2 (10)	0.2 (9)
Drinking status			
Non-drinker	49.6 (5,456)	24.2 (1,240)	71.8 (4,216)
Drinker	50.3 (5,538)	75.8 (3,888)	28.1 (1,650)
Missing	0.1 (8)	0.1 (3)	0.1 (5)
History of stroke	2.0 (215)	2.5 (128)	1.5 (87)
History of ACS	1.3 (144)	2.3 (116)	0.5 (28)
History of cancer	7.6 (839)	8.0 (410)	7.3 (429)
Cardiometabolic factors			
Body mass index, kg/m^2^	23.3 (3.4)	23.8 (3.1)	22.8 (3.5)
Presence of hypertension,^a^ %	43.8 (4,819)	50.6 (2,597)	37.8 (2,222)
Antihypertensive medications, %	28.3 (3,114)	32.1 (1,648)	25.0 (1,466)
Presence of diabetes,^b^ %	10.8 (1,193)	15.4 (789)	6.9 (404)
Antidiabetic medications, %	6.3 (697)	9.5 (485)	4.4 (259)
Presence of lipid abnormalities,^c^ %	48.3 (5,312)	49.5 (2,541)	47.2 (2,771)
Cholesterol-lowering medications, %	18.7 (2,058)	14.8 (761)	22.1 (1,297)

ACS, acute coronary syndrome.

All variables are expressed as means (standard deviations) for continuous variables or percentages (numbers) for categorical variables.

^a^Hypertension was defined as systolic blood pressure ≥140 mm Hg, diastolic blood pressure ≥90 mm Hg, or the use of antihypertensive medications.

^b^Diabetes was defined as fasting blood glucose ≥126 mg/dL, hemoglobin A1c ≥6.5% (National Glycohemoglobin Standardization Program), or the use of antidiabetic medications.

^c^Lipid abnormalities was defined as a low-density lipoprotein cholesterol level ≥160 mg/dL, high-density lipoprotein cholesterol level <40 mg/dL, triglyceride level ≥150 mg/dL, or use of cholesterol-lowering medications.

## STRENGTHS AND LIMITATIONS

The greatest strength of this cohort is the availability of genomic and metabolomic information for all the participants, which included 11,002 individuals from the general population. Optimization of the sample collection and pretreatment processes enabled us to maximize the accuracy of metabolome measurements compared with other platforms. Second, this cohort is particularly valuable as a large-scale multi-omics database in Japan and East Asia and will provide evidence that considers differences in genetic, lifestyle, and social backgrounds. As a relatively new cohort, it also provides up-to-date evidence of recent lifestyles and medical conditions. The high accuracy of tracking the outcomes of CVDs, cancer, death, and disability is another strength of this study. The follow-up rates were high, with only 147 cases of transfer to other cities as of October 2021. In all the follow-up surveys, blood and urine metabolome analyses were performed for all patients, providing longitudinal and significant data over time. A broad range of health-related items could be assembled, such as sensory and respiratory functions, because this study was coupled with health check-up programs offered by the government and workplace. The database also contains common test parameters and basic lifestyle habits for a wide range of social factors, which allows for the investigation beyond the biological etiology to elucidate socio-epidemiological insights using multi-omics data. In addition, the TMCS focuses on the assessment of environmental exposure, including new chemical substances and heavy metals, making it a platform that can be used for environmental epidemiology. Finally, all questionnaires were paired with face-to-face interviews conducted by trained personnel to minimize the occurrence of missing data and reduce misclassification. Validation against gold standards was performed on a subset of the population to increase the reliability of the self-reported status of medication use, physical activity level, and dietary intake.

One limitation of this study is its regional representativeness. One of four participants was recruited from workplaces that were willing to participate, while three of four were recruited from the local health municipal check-up examinees; thus, the selection was not completely random and might have attracted a healthier population. However, the health check-up program conducted by the municipality was open to all residents, and nine of 10 residents who participated in the program agreed to participate in the TMCS. Thus, it can be assumed that local representation is high, especially for the elderly (mean age of 63.3 years at baseline) recruited using the regional method. Another limitation is that Tsuruoka City is located in a relatively rural area; therefore, generalizability to an urban area may be limited, depending on the research topic. However, the influence of differences in the regional characteristics is likely to be limited with respect to the integrated approach to molecular profiling.

In conclusion, we will strive for a more accurate follow-up, expand our multi-omics data, and contribute to the realization of precision prevention in Japan and East Asia as a well-established multi-omics platform.

## SUPPLEMENTARY MATERIAL

eFigure 1. Flowchart of the participant selection process

## References

[1] Rattray NJW, Deziel NC, Wallach JD, . Beyond genomics: understanding exposotypes through metabolomics. Hum Genomics. 2018;12:4. 10.1186/s40246-018-0134-x 29373992 PMC5787293

[2] Assi N, Fages A, Vineis P, . A statistical framework to model the meeting-in-the-middle principle using metabolomic data: application to hepatocellular carcinoma in the EPIC study. Mutagenesis. 2015;30:743–753. 10.1093/mutage/gev045 26130468 PMC5909887

[3] Vineis P, Demetriou CA, Probst-Hensch N. Long-term effects of air pollution: an exposome meet-in-the-middle approach. Int J Public Health. 2020;65:125–127. 10.1007/s00038-019-01329-7 31927609

[4] Vineis P, van Veldhoven K, Chadeau-Hyam M, Athersuch TJ. Advancing the application of omics-based biomarkers in environmental epidemiology. Environ Mol Mutagen. 2013;54:461–467. 10.1002/em.21764 23519765

[5] Wild CP. The exposome: from concept to utility. Int J Epidemiol. 2012;41:24–32. 10.1093/ije/dyr236 22296988

[6] Wishart DS. Metabolomics for investigating physiological and pathophysiological processes. Physiol Rev. 2019;99:1819–1875. 10.1152/physrev.00035.2018 31434538

[7] Hirayama A, Sugimoto M, Suzuki A, . Effects of processing and storage conditions on charged metabolomic profiles in blood. Electrophoresis. 2015;36:2148–2155. 10.1002/elps.201400600 25820922

[8] Guasch-Ferré M, Bhupathiraju SN, Hu FB. Use of metabolomics in improving assessment of dietary intake. Clin Chem. 2018;64:82–98. 10.1373/clinchem.2017.272344 29038146 PMC5975233

[9] Posma JM, Garcia-Perez I, Frost G, . Nutriome-metabolome relationships provide insights into dietary intake and metabolism. Nat Food. 2020;1:426–436. 10.1038/s43016-020-0093-y 32954362 PMC7497842

[10] World Health Organization. Integrated care for older people (ICOPE): guidance for person-centred assessment and pathways in primary care, https://www.who.int/publications/i/item/WHO-FWC-ALC-19.1; 2019. Accessed 31.05.2023.

[11] Beard JR, Officer A, de Carvalho IA, . The World report on ageing and health: a policy framework for healthy ageing. Lancet. 2016;387:2145–2154. 10.1016/S0140-6736(15)00516-4 26520231 PMC4848186

[12] Sata M, Kakino A, Hirata A, . Serum modified high-density lipoprotein and risk of atherosclerotic cardiovascular disease in a Japanese community-based nested case-control study. Eur J Prev Cardiol. 2022;29:e193–e195. 10.1093/eurjpc/zwab142 34472612

[13] Sata M, Okamura T, Harada S, . Association of the estimated coronary artery incidence risk according to the Japan Atherosclerosis Society Guidelines 2017 with cardio-ankle vascular index. J Atheroscler Thromb. 2021;28:1266–1274. 10.5551/jat.58719 33678765 PMC8629702

[14] Harada S, Sata M, Matsumoto M, . Changes in smoking habits and behaviors following the introduction and spread of heated tobacco products in Japan and its effect on FEV(1) decline: a longitudinal cohort study. J Epidemiol. 2022;32:180–187. 10.2188/jea.JE20210075 34657910 PMC8918621

[15] Japanese Committee for Clinical Laboratory Standards. https://www.jccls.org/ (in Japanese); 2023. Accessed 31.05.2023.

[16] Sasaki M, Miyagawa N, Harada S, . Dietary patterns and their associations with intermediate age-related macular degeneration in a Japanese population. J Clin Med. 2022;11(6):1617. 10.3390/jcm11061617 35329943 PMC8955354

[17] Treacy D, Hassett L. The short physical performance battery. J Physiother. 2018;64:61. 10.1016/j.jphys.2017.04.002 28645532

[18] Shibuki T, Iida M, Harada S, . The association between sleep parameters and sarcopenia in Japanese community-dwelling older adults. Arch Gerontol Geriatr. 2023;109:104948. 10.1016/j.archger.2023.104948 36764202

[19] Folstein MF, Folstein SE, McHugh PR. “Mini-mental state”. A practical method for grading the cognitive state of patients for the clinician. J Psychiatr Res. 1975;12:189–198. 10.1016/0022-3956(75)90026-6 1202204

[20] Nasreddine ZS, Phillips NA, Bédirian V, . The Montreal Cognitive Assessment, MoCA: a brief screening tool for mild cognitive impairment. J Am Geriatr Soc. 2005;53:695–699. 10.1111/j.1532-5415.2005.53221.x 15817019

[21] Fujiwara Y, Suzuki H, Yasunaga M, . Brief screening tool for mild cognitive impairment in older Japanese: validation of the Japanese version of the Montreal Cognitive Assessment. Geriatr Gerontol Int. 2010;10:225–232. 10.1111/j.1447-0594.2010.00585.x 20141536

[22] Imaeda N, Goto C, Sasakabe T, . Reproducibility and validity of food group intake in a short food frequency questionnaire for the middle-aged Japanese population. Environ Health Prev Med. 2021;26:28. 10.1186/s12199-021-00951-3 33653279 PMC7923820

[23] Tokudome Y, Goto C, Imaeda N, . Relative validity of a short food frequency questionnaire for assessing nutrient intake versus three-day weighed diet records in middle-aged Japanese. J Epidemiol. 2005;15:135–145. 10.2188/jea.15.135 16141632 PMC7851066

[24] Fukai K, Harada S, Iida M, . Metabolic profiling of total physical activity and sedentary behavior in community-dwelling men. PLoS One. 2016;11:e0164877. 10.1371/journal.pone.0164877 27741291 PMC5065216

[25] Matsumoto M, Harada S, Iida M, . Validity assessment of self-reported medication use for hypertension, diabetes, and dyslipidemia in a pharmacoepidemiologic study by comparison with health insurance claims. J Epidemiol. 2021;31:495–502. 10.2188/jea.JE20200089 33361656 PMC8328856

[26] Harada S, Hirayama A, Chan Q, . Reliability of plasma polar metabolite concentrations in a large-scale cohort study using capillary electrophoresis-mass spectrometry. PLoS One. 2018;13:e0191230. 10.1371/journal.pone.0191230 29346414 PMC5773198

[27] Hirayama A, Nakashima E, Sugimoto M, . Metabolic profiling reveals new serum biomarkers for differentiating diabetic nephropathy. Anal Bioanal Chem. 2012;404:3101–3109. 10.1007/s00216-012-6412-x 23052862

[28] Hirayama A, Tomita M, Soga T. Sheathless capillary electrophoresis-mass spectrometry with a high-sensitivity porous sprayer for cationic metabolome analysis. Analyst. 2012;137:5026–5033. 10.1039/c2an35492f 23000847

[29] Hirayama A, Kami K, Sugimoto M, . Quantitative metabolome profiling of colon and stomach cancer microenvironment by capillary electrophoresis time-of-flight mass spectrometry. Cancer Res. 2009;69:4918–4925. 10.1158/0008-5472.CAN-08-4806 19458066

[30] Ishibashi Y, Harada S, Takeuchi A, . Reliability of urinary charged metabolite concentrations in a large-scale cohort study using capillary electrophoresis-mass spectrometry. Sci Rep. 2021;11:7407. 10.1038/s41598-021-86600-9 33795760 PMC8016858

[31] Hirayama A, Ishikawa T, Takahashi H, . Quality control of targeted plasma lipids in a large-scale cohort study using liquid chromatography-tandem mass spectrometry. Metabolites. 2023;13(4):558. 10.3390/metabo13040558 37110217 PMC10146188

[32] Sakurai-Yageta M, Kumada K, Gocho C, . Japonica Array NEO with increased genome-wide coverage and abundant disease risk SNPs. J Biochem. 2021;170:399–410. 10.1093/jb/mvab060 34131746 PMC8510329

[33] Tadaka S, Katsuoka F, Ueki M, . 3.5KJPNv2: an allele frequency panel of 3552 Japanese individuals including the X chromosome. Hum Genome Var. 2019;6:28. 10.1038/s41439-019-0059-5 31240104 PMC6581902

[34] Delaneau O, Zagury JF, Marchini J. Improved whole-chromosome phasing for disease and population genetic studies. Nat Methods. 2013;10:5–6. 10.1038/nmeth.2307 23269371

[35] Bycroft C, Freeman C, Petkova D, . The UK Biobank resource with deep phenotyping and genomic data. Nature. 2018;562:203–209. 10.1038/s41586-018-0579-z 30305743 PMC6786975

[36] 1000 Genomes Project Consortium, Auton A, Brooks LD, . A global reference for human genetic variation. Nature. 2015;526:68–74. 10.1038/nature15393 26432245 PMC4750478

[37] Ohmomo H, Harada S, Komaki S, . DNA methylation abnormalities and altered whole transcriptome profiles after switching from combustible tobacco smoking to heated tobacco products. Cancer Epidemiol Biomarkers Prev. 2022;31:269–279. 10.1158/1055-9965.EPI-21-0444 34728466 PMC9398167

[38] Hachiya T, Furukawa R, Shiwa Y, . Genome-wide identification of inter-individually variable DNA methylation sites improves the efficacy of epigenetic association studies. NPJ Genom Med. 2017;2:11. 10.1038/s41525-017-0016-5 29263827 PMC5677974

[39] Komaki S, Ohmomo H, Hachiya T, . Evaluation of short-term epigenetic age fluctuation. Clin Epigenetics. 2022;14:76. 10.1186/s13148-022-01293-9 35681206 PMC9185970

[40] Ministry of Health, Labour and Welfare. The National Health and Nutrition Survey. https://www.mhlw.go.jp/bunya/kenkou/eiyou/h24-houkoku.html; 2012. Accessed 31.05.2023.

