# Supplementary figures and images for: Study Profile of the Tsuruoka Metabolomics Cohort Study (TMCS)
Source: J Epidemiol. 2024 Aug 5;34(8):393–401. doi: 10.2188/jea.JE20230192 (PMC11230875; doi:10.2188/jea.JE20230192)

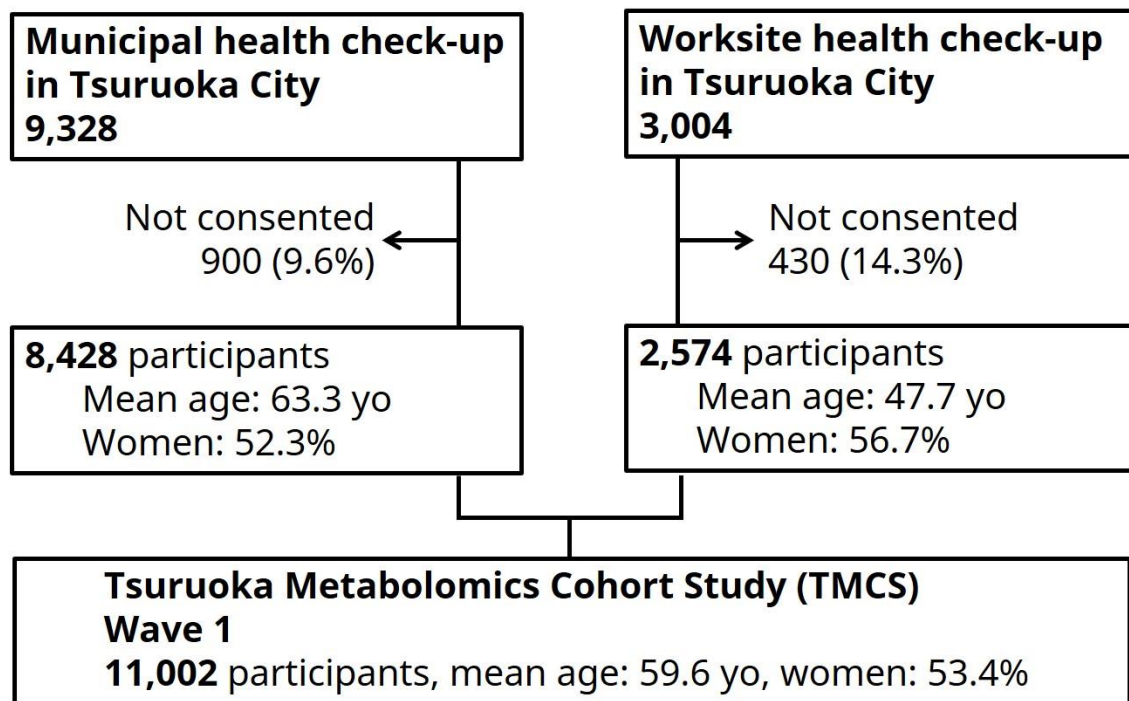

**eFigure 1.** Flowchart of the participant selection process

Supplement: Supplementary file 1 [file je-34-393-s001.pdf]
